# Data Base Management System for Lymphatic Filariasis - A Neglected Tropical Disease

**DOI:** 10.1371/journal.pone.0039970

**Published:** 2012-07-05

**Authors:** Suryanaryana Murty Upadhyayula, Srinivasa Rao Mutheneni, Madhusudhan Rao Kadiri, Sriram Kumaraswamy, Sarat Chandra Babu Nelaturu

**Affiliations:** 1 Biology Division, CSIR-Indian Institute of Chemical Technology, Hyderabad, India; 2 Centre for Development of Advanced Computing, Hyderabad, India; University of Massachusetts Medical School, United States of America

## Abstract

**Background:**

Researchers working in the area of Public Health are being confronted with large volumes of data on various aspects of entomology and epidemiology. To obtain the relevant information out of these data requires particular database management system. In this paper, we have described about the usages of our developed database on lymphatic filariasis.

**Methods:**

This database application is developed using Model View Controller (MVC) architecture, with MySQL as database and a web based interface. We have collected and incorporated the data on filariasis in the database from Karimnagar, Chittoor, East and West Godavari districts of Andhra Pradesh, India.

**Conclusion:**

The importance of this database is to store the collected data, retrieve the information and produce various combinational reports on filarial aspects which in turn will help the public health officials to understand the burden of disease in a particular locality. This information is likely to have an imperative role on decision making for effective control of filarial disease and integrated vector management operations.

## Introduction

Lymphatic filariasis (LF) is the second most common vector borne disease followed by malaria and is found in over 80 tropical and subtropical countries. Filariasis is one of the leading causes of disability infecting 120 million individuals and 1.3 billion people are at risk of infection [1]. To combat this disease, World Health Assembly has passed a resolution to eliminate LF by the year 2020, for which Global Programme for Elimination of Lymphatic Filariasis (GPELF) began during 1999 in all the LF endemic countries. The aim of GPELF is to interrupt transmission of the parasite using annual mass drug administration (MDA) by single dose of diethylcarbamazine citrate (DEC) to prevent LF-related disability. There are about 381 million people who have received this treatment in 42 countries [1,2,3&4].

About one third of filarial cases world-wide are found in India [5]. This disease is endemic in 18 states and the Union Territories of India. Approximately 420 million people reside in endemic areas and 48.11 million are infected. Mortality is uncommon, whereas morbidity associated with this infection can be considerable and lifelong. Because of these factors, LF was a neglected disease and lacked the attention of planners and successive governments. The rural and urban areas of India suffer with lack of adequate antifilarial measures and it is estimated 52.93 million populations is protected by the National Filaria Control Programme (NFCP), Government of India.

In India, MDA with DEC was launched as a pilot project in 13 districts of 7 states in the year 1996, later it has been implemented to all endemic states/districts of the country [6]. The MDA campaign in 2005 covered a population of 463 million using DEC alone and 17.34 million with DEC plus albendazole combination [1]. Out of 23 districts of Andhra Pradesh, India, 16 districts are more prevalent for lymphatic filariasis and MDA program has been continuing since 2004, covering a population of 54 million [7]. According to Ministry of Health & Family Welfare, Govt. of India the microfilaria rates of Andhra Pradesh are gradually declining and are as follows: in the year 2000-1.85%, 2001-1.36%, 2002-1.14%, 2003-0.78% and 2004-0.57%.

To eliminate LF by the year 2020, it is necessary to understand the consequences of this disease burden, planning the control policies in more appropriate manner such as proper epidemiological and entomological surveillance studies and participation in MDA programs. In order to ensure a control strategy to be more effective and appropriate, the collection and compilation of data (epidemiological, entomological and socioeconomic field studies) needs to be of a high standard followed by proper approach for data analysis and diseases management [3], [4].

Over the past several years, advances in public health have enabled the researchers to carry out epidemiological data collection and management in a high throughput fashion. The success of an epidemiological study depends on many factors including the complexities in management of epidemiological data and database support [3]. Development of database is one of the fundamental objectives of the data management and it is one of the end products of the process. The data management is processing of the data from various sources into a central database as well as the process of data quality [8]. A set of logically sequenced operational components comprising the data management system are: data collection, entry, editing, monitoring, storage, sorting, linking, grouping, retrieval and analysis of data [3], [9]. Moreover, these data are used in studies of public health diseases which are complex and consist of various micro and macro parameters. The ultimate goal of databases is to present the information contained in the data so that it improves knowledge of public health processes [10]. Innovative Vector Control Consortium has identified that Information Technology and its tools helps to improve the vector control mechanisms and leads to reduce the burden of vector borne disease [11]. As there is no public health database scheme (consisting of epidemiological, entomological, meteorological and socioeconomic data) on lymphatic filariasis we have developed a complete and flexible database system for potential control of this neglected tropical disease. This database is a web-based interface that integrates good organization, simple and user friendly accessibility, data security, multi-user support system, generation of various combinatorial reports, data analysis through data mining tools and supports GIS applications.

## Results

**Figure 1:**
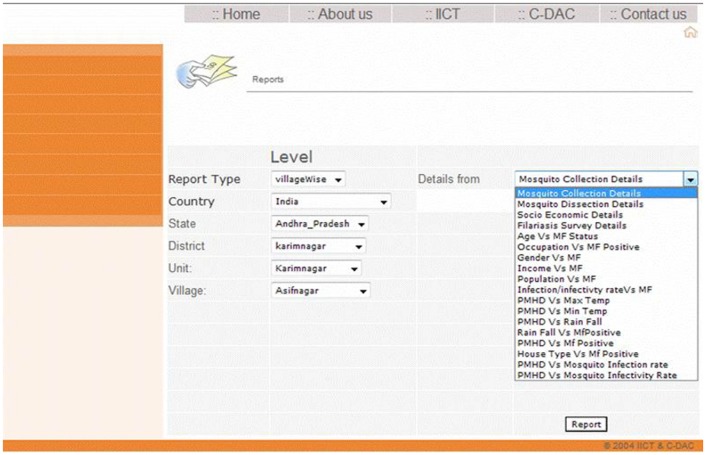
Screen shot of combinational reports.

Dilating from the home page of filariasis database which explains about the disease traversing through various modules viz., area details module, meteorological details module etc., interactive facilitation to enter and retrieve various details has been manifested in Table-1. The reports generated in PDF and HTML format hasn’t been confined to a single stream but subjected to get through all possible combinations of the parameters engrafted in the database as depicted in Figure-1. Thus a user gets to know ‘age-wise’, ‘gender-wise’ positive filarial cases in the given locality. In entomological footings, ‘infection-rate’ or ‘infectivity’ based authentication of ‘microfilaria carriers’ in the region could be arrayed. Modelling the interplay of the analyzed candidatures in this effort, elaborates its extensive utilization in real-time. The minimalism of use of this application could be understood well only through proper trainings or workshops.

**Table 1:** Conceptual modules of the filariasis database schema.

Module	Information
Filariasis Home Page	This is the starting page of this filariasis database application. This gives brief introduction about filariasis, bibliographic information, related journals, and various links to online resources of filariasis and also provides text fields to enter login details.
Clinical Information	Filariasis disease symptoms, signs, risk factors, disease prevention methods, treatment, mosquito control details are provided.
User Management Module	This module provides facility to create/update/view/delete a user. It also provides facilities to user authentication. For authenticated users it gives privileged access to filariasis database management system. This module is accessible to administrator only.
Area Details Module	This is used to register/enter the area (country/state/district/unit/mandal/village) details. The scientific users can navigate through this interface only.
Meteorological Details Module	This interface gives the facility to user to enter the monthly meteorological details like maximum/minimum temperature, total rainfall, relative humidity, and wind speed of a particular area are entered through this module.
Socio Economic Details Module	This module provides facility to enter socio economic status of a person.
Mosquito collection Module	Provides information about Mosquito Collection Details. Also contains hyper links to various sub module of Mosquito Collection Details module.
Mosquito dissection Module	This interface provides the functionality for entering mosquito dissection related information.
Mass blood survey module	Through this interface user can enter the details of mass blood survey collected from the different villages corresponding to individual family. Also contains hyper links to various sub module of mass blood survey module.
Filariasis survey module	Through this interface user can enter the details of filariasis survey (i.e. filariasis affected people) collected from the different villages corresponding to individual family.
Reports module	This interface helps in navigating between different reports for different modules based on selected village.
	The Reports for this application also has been generated in the form of combinational report of distinct factors. There are two report formats i.e.
	1. PDF format.
	2. HTML format.
	The reports has been categorized on the following basis :
	Village-wise.
	District-wise.

### Testing

The filariasis database was tested by using the field collected data from the study areas. The data was entered in all the modules and generated various reports including combinational reports like microfilaria parasite prevalence versus gender, education status, occupation, house structure etc. are provided accurately.

The Data validation has been done at two levels one at web application level by providing user input fields check using JAVASCRIPT and second at database level by using domain level constraints and integrity constraints, these process assures the quality of the data in the database. The default transaction isolation level i.e., READ_COMMITTED is used in the database application. Hence, no unwanted reads will not occur. This default transaction level suffices the purpose of data integrity with respect to filariasis database.

### Usability and Data Access Permissions

Filariasis database is developed for public health authorities (people who are handling large volumes of data), state/central institutes and laboratories working on epidemiological studies of filariasis. The database schema covers all relevant information on filariasis and becomes a very versatile tool for management of the filariasis diseases.

The LF database can be used from multiple sites on the internet or LAN for entering the data in the web application. Since the database is always behind the web application deployed on web server, the consolidation of the entire data entry request from different sites is being done by web server and sent to database server. All constraints are being used in the database and applied to the ready data to be entered in the database. The data audit can be done by maintaining the transaction logs at DBMS level that can be only accessed by the Database Administrator (DBA). Thus, at the time of user signed on to perform data entry, the appropriate data entry restrictions could be applied as described by the database administrator.

### Implementation of Database

**Figure 2:**
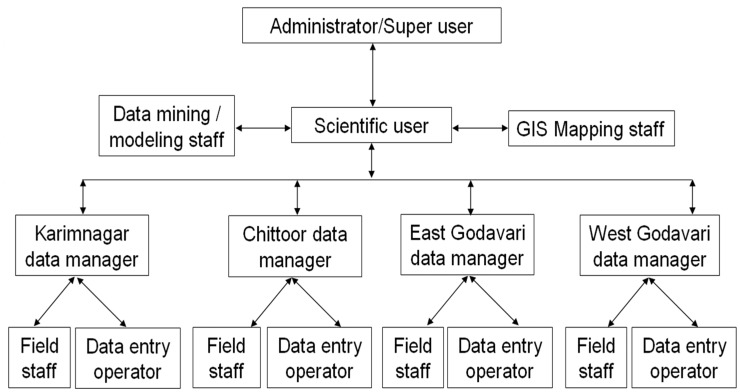
Data management and organizational frame work in study districts of Andhra Pradesh, India.

Prior to the implementation of the database, an organizational framework (Figure-2) was established to provide the infrastructure from which the database system could be accessed. Imposition of a file management structure creates standardization, a key factor in producing quality data, which is particularly important when there are multiple sites for data entry. To light upon, in the first phase the database has already been implemented successfully in Karimnagar, Chittoor, East and West Godavari, district of Andhra Pradesh. All the health officials of these districts were trained in phase wise manner and handed over the database to the department of health Government of Andhra Pradesh for its implementation (http://www.iictindia.org/IICT_WEB/Filariasis.htm). During regular interaction with the district health authorities in connection with the performance of the database in day to day use we received very encouraging reports from the end users.

## Discussion

The development of database for epidemiological studies is tedious process and it provides large numbers of observations, collected during study period. The database is able to deal with more complex form of designs and linkages between the variables for data entry comparisons. The quality control functionality is superior and the output generated demonstrates the validity of the data and provides public health users with quality assurance. Therefore it is more advantageous to users during epidemiological, entomological studies of filariasis. As the database covers various modules, the final output has been designed as combinatorial reports interlinking various parameters to coalesce all possible priorities from the user.

**Figure 3:**
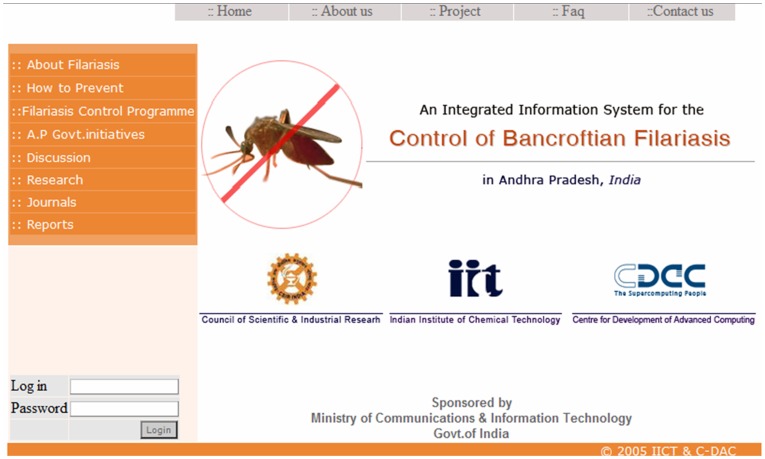
Filariasis database home page and user login details.

The benefits of the database are to provide accuracy, efficiency and ability to provide useful analysis of the data. In this context the database has user-friendly interface and simple usage mainly through diversified data entry forms like entomology (mosquito collection and dissection details) epidemiology (mass blood survey for LF**,** acute and chronic LF cases) meteorological and socioeconomic details. Each user needs a username and a password to access filariasis database (Figure-3). After log in user can enter the data, view and modify the data and also delete information from the database server.

The automated double-entry check is a key component of the database. During the second data entry, an automatic comparison is made between the values just entered for a particular field with the value entered for that field during the first entry. Users are notified of any discrepancies between the two datasets and are prompted to select the correct value or override the data and synchronize the two data files. User can also check any missing data entered. In addition to collation of data in to the database, data analysis (data mining) and spatial mapping tools are highly compatible in this database.

### Future Directions

Beside use of chemotherapeutic drugs (using albendazole with diethylcarbamazine or albendazole with ivermectin), proper disease surveillance and disease management would ably suppress any vector borne disease in a more effective way. The best example is the implementation of a web-based National Anti Malaria Management Information System (NAMMIS), throughout India. Similar kind of approach is utilized in our study, to develop a database on lymphatic filariasis. This database is user friendly and for quick report generation on epidemiological and other related data. User can include new addition of data to generate many combinational reports on various input parameters. These reports would help the health officials to understand the overall spectrum of disease and combat the disease in an effective way. The developed database on filariasis can be utilized for management of other such vector borne diseases, after proper incorporation of various relevant parameters. The most important future directions are phase wise pilot scale implementation through health management network system throughout the country.

## Conclusions

A good database provides high reliability and accurate information in the form of output or the queries raised by the user. The output of report should be simple to understand and easy to interpret. In most of the epidemiological studies there is a serious knowledge gap in understanding the principles and practices of data management which can be overcome by implementing database management system [3]. The developed web enabled database on filariasis is very user friendly and provides accurate and reliable data that plays an important role in filariasis disease management.

## Materials and Methods

### Data Collection

The study was undertaken in 120 villages from Karimnagar, Chittoor, East and West Godavari districts of Andhra Pradesh during 2004 to 2007. The study received ethical clearance from the Ethical Committee which was constituted in our institute (Indian Institute of Chemical Technology) affiliated to Ministry of Science and Technology, Govt of India. This ethical committee has approved to carry out the research work. Before commencing investigations, the local authorities and the residents of the selected villages were informed about the proposed study and obtained their written consent. The respondents were selected randomly from all parts of the village. During the survey epidemiological (to asses the microfilaria {MF} infection), entomological and socioeconomic data were collected simultaneously by involving two sets of health volunteers. The socioeconomic details were collected only from people who were subjected to epidemiological study. Information on family characteristics with a possible influence on filariasis like sex, age, use of mosquito avoidance measures (like bed net, coils etc.), awareness on filariasis, number of children in a family, place of residence, family’s monthly income, house structure (living in a hut, thatched, tiled and reinforced cement concrete {RCC}), education details, occupation information, vector breeding habitats, participation of MDA program etc., was collected through interviewing the head of the family and other family members using a structured questionnaire. The questionnaire was composed according to local requirements and appropriateness.

### Database Design

Designing the skeleton of database is an important task in development of the application. The important aspects in designing the database consist of development of questionnaire, data collection forms, data entry screens and data tables which have been incorporated into the database, normalisation of data across tables, to prioritize important fields and to create a network of the tables within the database. Relational database has been used for development of filariasis database; it is the most popular model among currently available database management system [12]. The relational database management system (RDBMS) is a reliable method to store the information without redundancy and retrieving large amounts of data, offering a blend of system performance and ease of implementation. RDBMS is a collection of tables that are linked together by keys to create relationships between data entities. The database delineated here, is an advanced representation of output data (based on input query: output in the form of combinational reports generated by merging of various fields) of filariasis. We have extensively discussed with researchers and computer professionals who actually work in the relevant fields about their needs and finally we have divided all the sources of information into eleven conceptual modules around which the filariasis database schema has been structured (Table-1).

### Database Architecture

**Figure 4:**
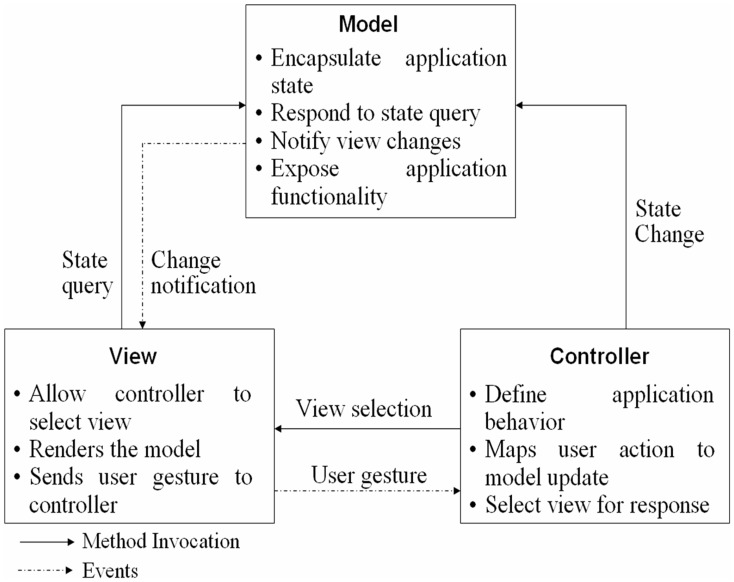
Model view controller architecture.

The architecture of a database refers to the manner in which the entries in a database are organized for archiving easy retrieval. The trial design being complex requires a database with numerous data entry forms, corresponding tables and specified inter-table relationships, hence model view controller (MVC) architecture (Figure-4) has been used for developing web based filariasis database. The main components of MVC are (i) models (maintain program data logics, stores the data like relational databases), (ii) views (presents the data on web pages) and (iii) controllers (handle events, determines the overall flow of the application). Struts, an open source framework used for developing J2EE web applications through MVC: a design pattern is a common coding skeletal framework on which database application has been employed for creating and processing web-based forms. This application is evolved with the MySQL as database at the backend.

### Data Security

Data security is also implemented in this database in the itinerary of the identification of the user’s log in. In the web-based interface, the user must be identified with a username and a password, both corresponding to those implemented in the local MySQL server. Similarly, the database server accepts connection requests only from the machine where web server is hosted. The database server can be configured to accept connections from a particular machine identified by its domain name or internet protocol (IP) address. Similarly, Secure transmission control protocol (TCP)/IP connections with secure shell (SSH) tunnel can use SSH to encrypt the network connection between clients and a MySQL server.

### Database Forms

**Figure 5:**
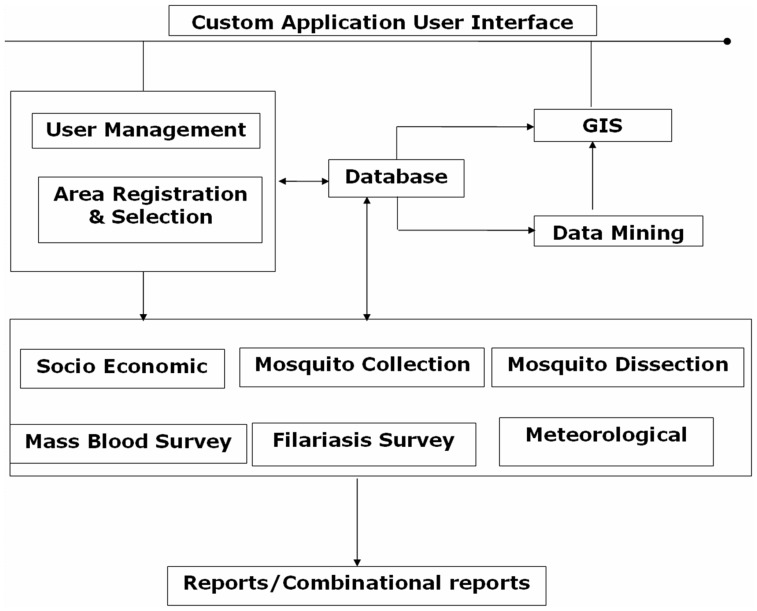
Data flow information of the filariasis database.

During disease surveillance studies precise enumeration of the quantity and type of data collected and this data is entered in the database. Data integrity was verified during the development of each work file created for analysis. Information from the database tables was compared with the documents to determine errors if any in the data which was incorporated in the database. Figure-5 shows the flow chart of information involved along this process.

- The flow diagram of information (Figure-5) starts with the registration of the user management module (Figure-S1). Based on the user registration details administrator provides the privileged access of filariasis database management system to the user.
- Followed by registration of new study area (Figure-S2) details (by State/district/unit/village wise) or selection of study area (by State/district/unit/village wise) from already registered list.
- The next step is selection of data entry forms. The user can enter the data in required forms such as socioeconomic (Figure-S3), mosquito collection (Figure-S4), dissection (Figure-S5), mass blood survey (Figure-S6), filariasis disease survey (Figure-S7) details and meteorological data (Figure-S8).
- The final step of the database is report generation, where the user can generate epidemiological, entomological, socioeconomic and meteorological reports. Apart from this, user can also produce the various kinds of reports by village/district wise also.
- Generation of combinational reports (Figure-1) is one of the main advantages of this database. The user can correlate the epidemiological data with entomological, meteorological and socioeconomic parameters and vice versa and thus assess the important parameter which contributes transmission or spreading of the disease.
- This kind of report assists public health officials for identifying the importance of micro and macro parameters which further helps in focusing more on those parameters for effective management of the disease.

## Supporting Information

Figure S1 **User management module of filariasis database.**
(TIF)

Figure S2 **Area details of the study area.**
(TIF)

Figure S3 **Socioeconomic details of filariasis database.**
(TIF)

Figure S4 **Mosquito collection details of filariasis database.**
(TIF)

Figure S5 **Mosquito dissection details of filariasis database.**
(TIF)

Figure S6 **Mass blood survey details of filariasis.**
(TIF)

Figure S7 **Filariasis disease survey details form.**
(TIF)

Figure S8 **Meteorological details form.**
(TIF)
