# Supplementary figures and images for: Data Base Management System for Lymphatic Filariasis - A Neglected Tropical Disease
Source: PLoS One. 2012 Jul 5;7(7):e39970. doi: 10.1371/journal.pone.0039970 (PMC3390335; doi:10.1371/journal.pone.0039970)

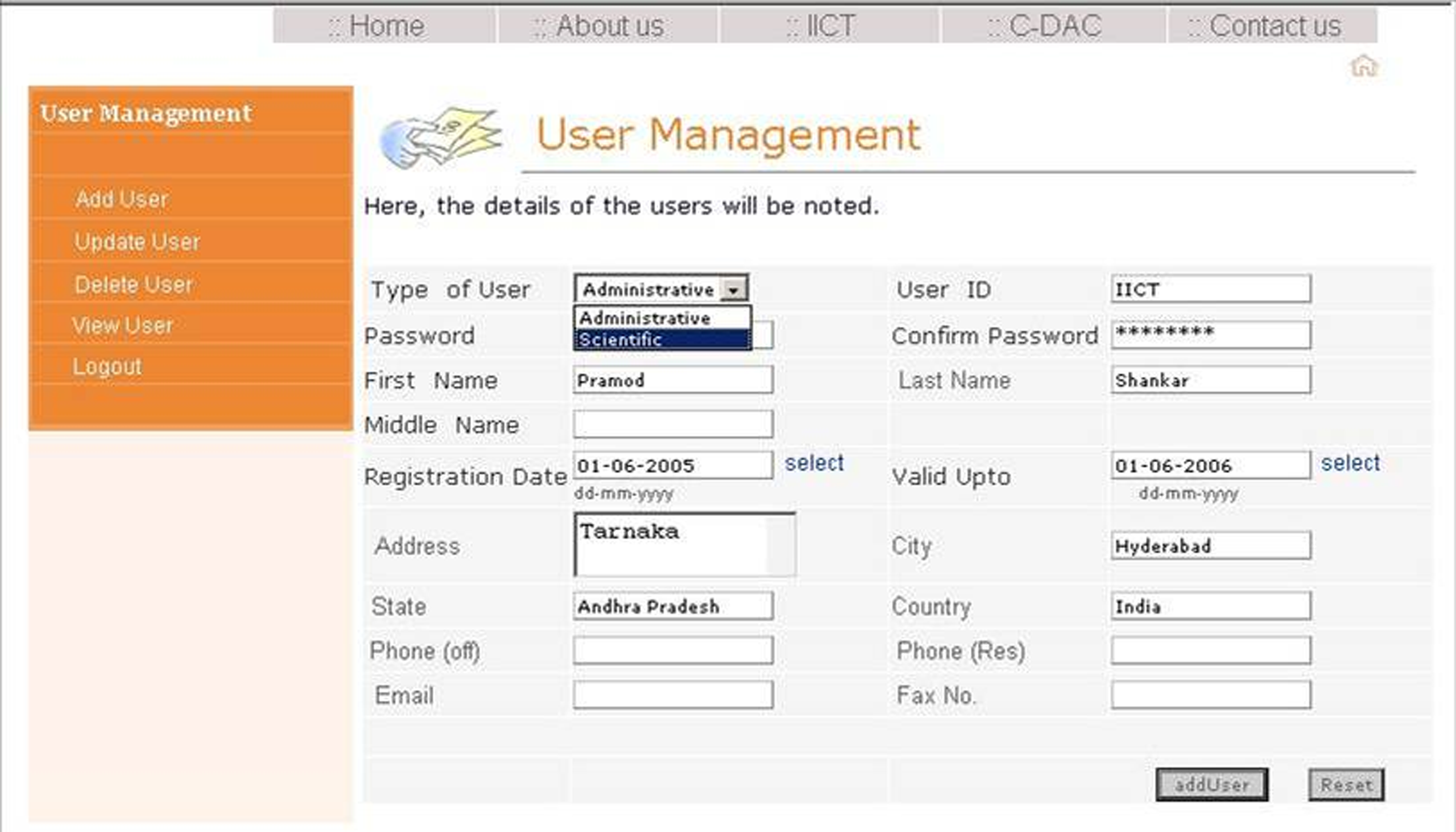

Supplement: Figure S1 — User management module of filariasis database. (TIF) [file pone.0039970.s001.tif]

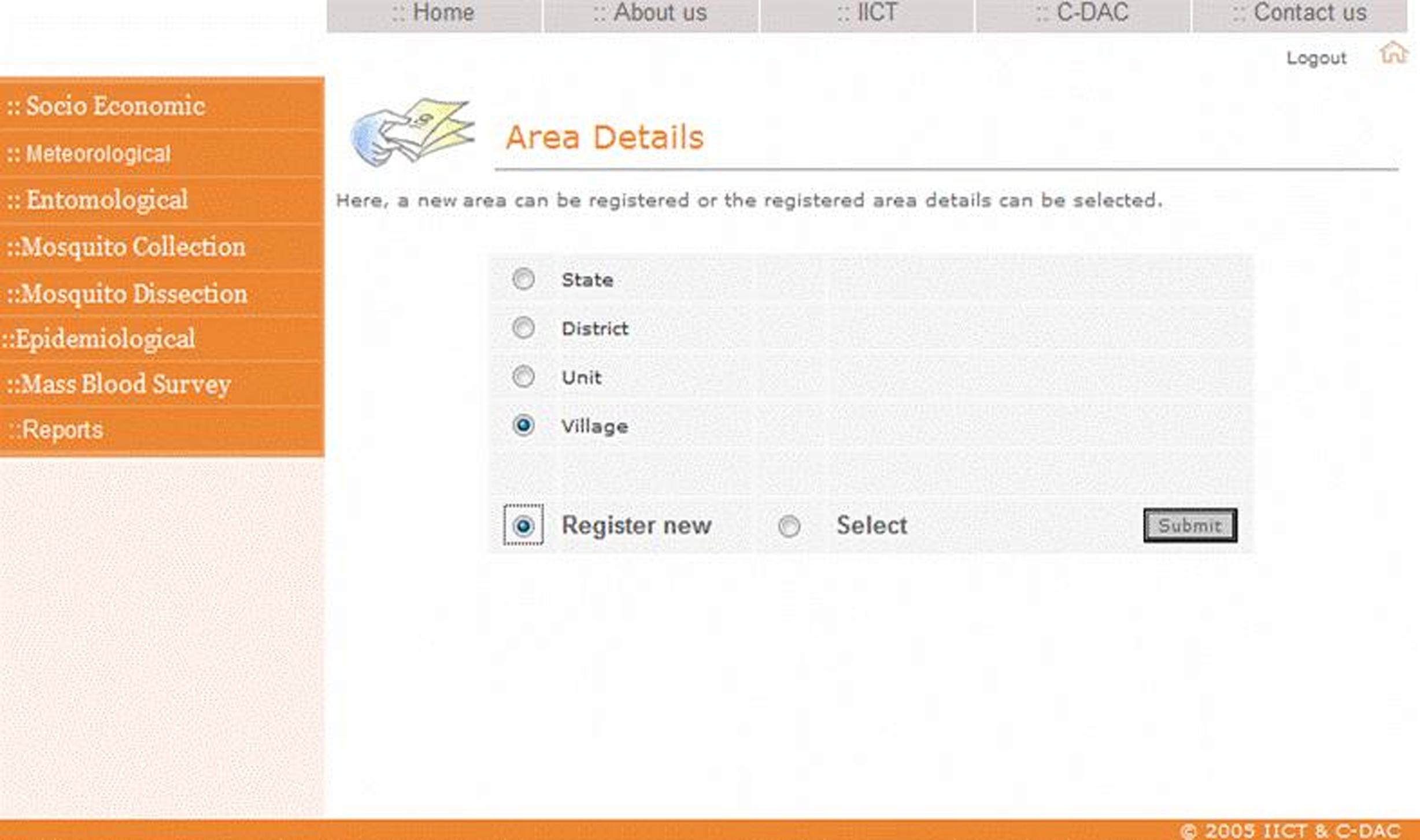

Supplement: Figure S2 — Area details of the study area. (TIF) [file pone.0039970.s002.tif]

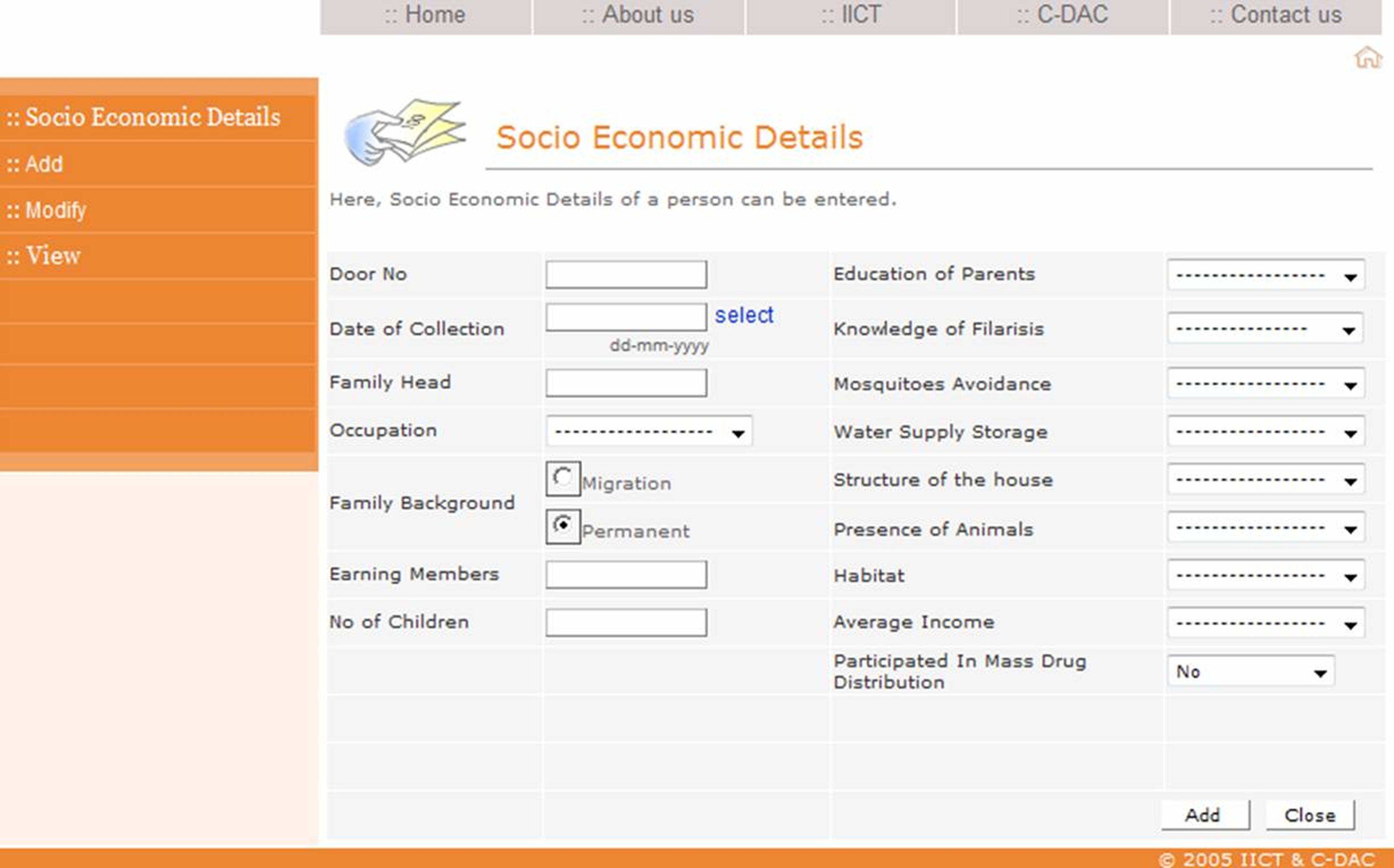

Supplement: Figure S3 — Socioeconomic details of filariasis database. (TIF) [file pone.0039970.s003.tif]

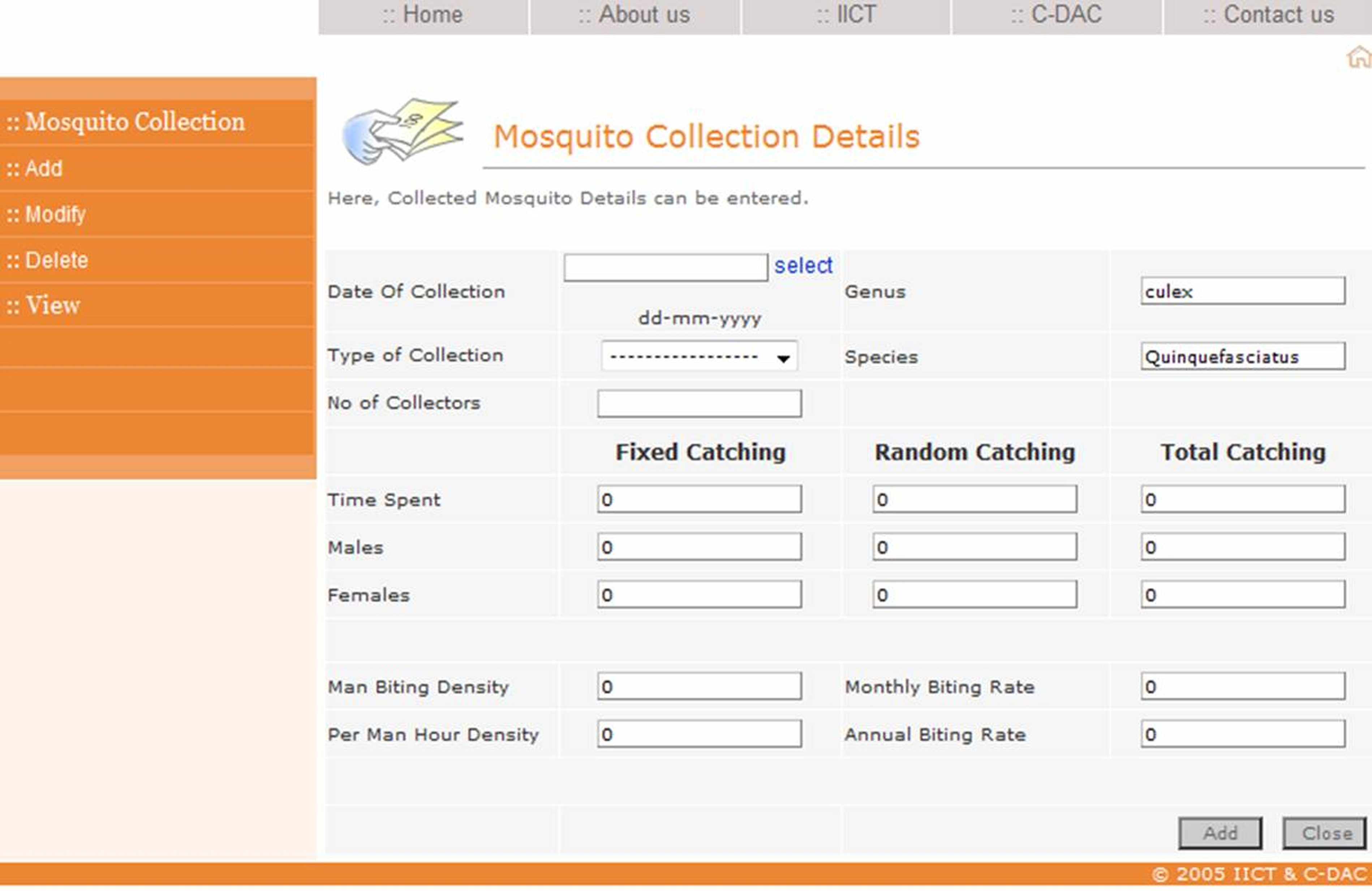

Supplement: Figure S4 — Mosquito collection details of filariasis database. (TIF) [file pone.0039970.s004.tif]

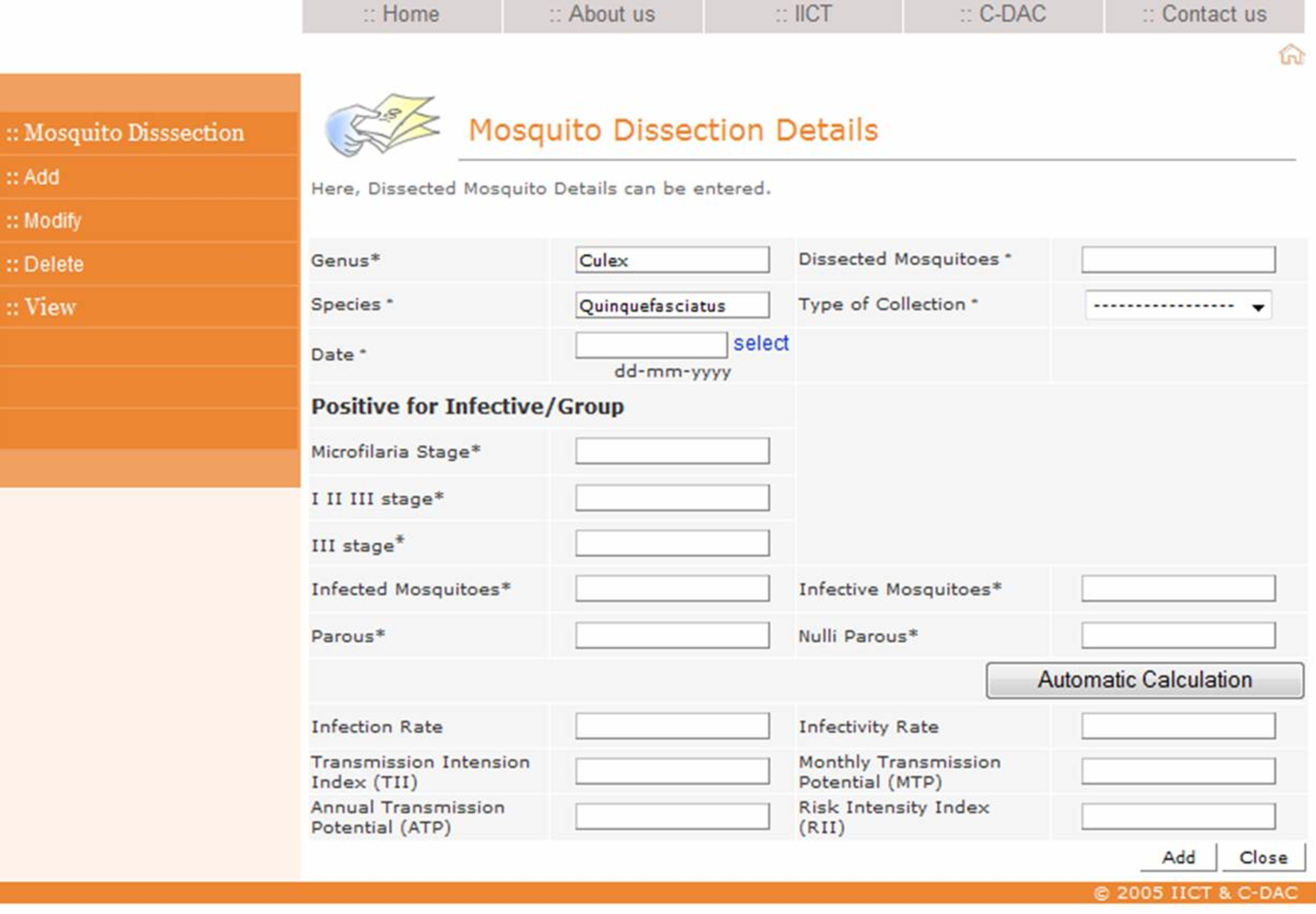

Supplement: Figure S5 — Mosquito dissection details of filariasis database. (TIF) [file pone.0039970.s005.tif]

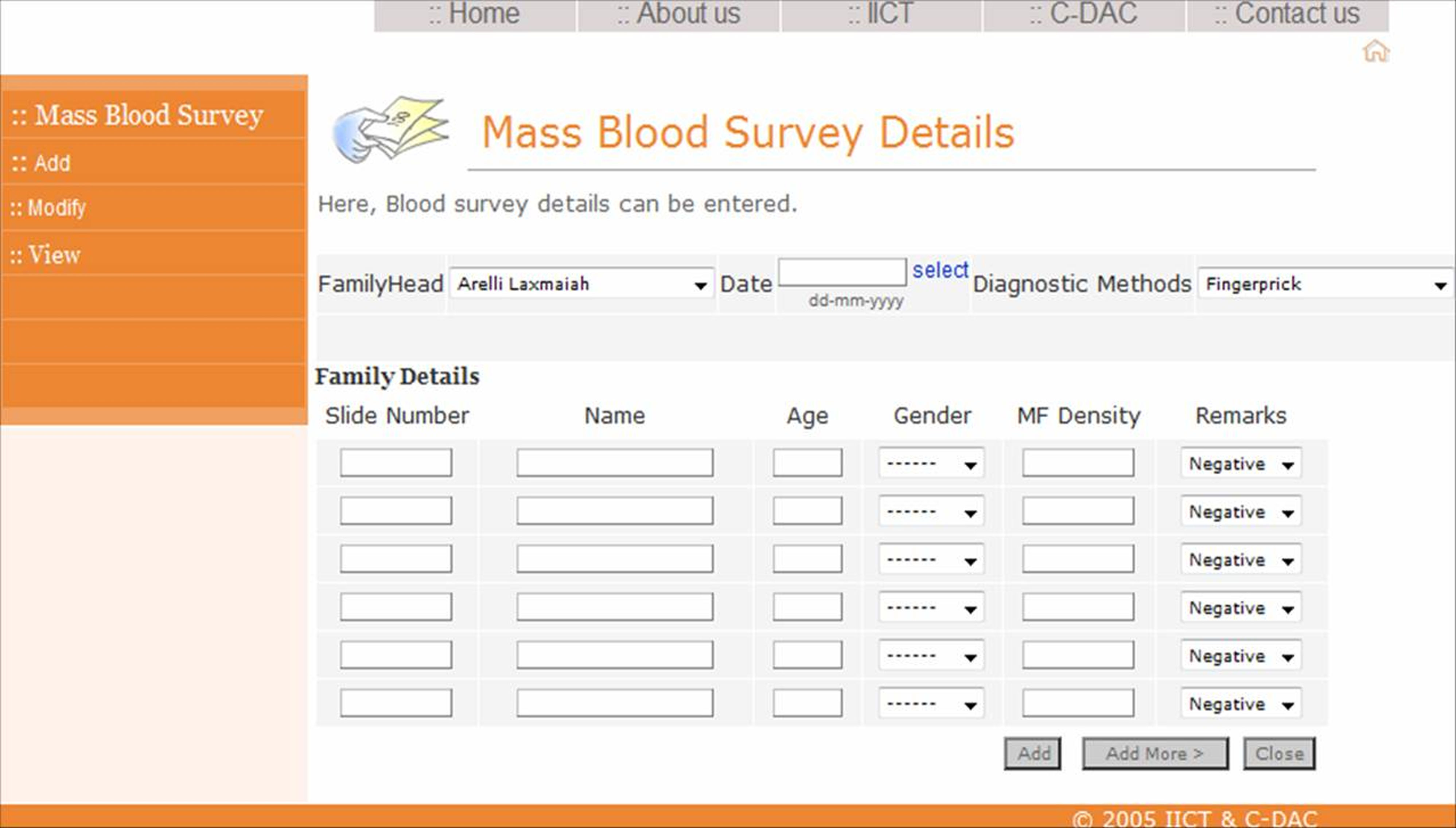

Supplement: Figure S6 — Mass blood survey details of filariasis. (TIF) [file pone.0039970.s006.tif]

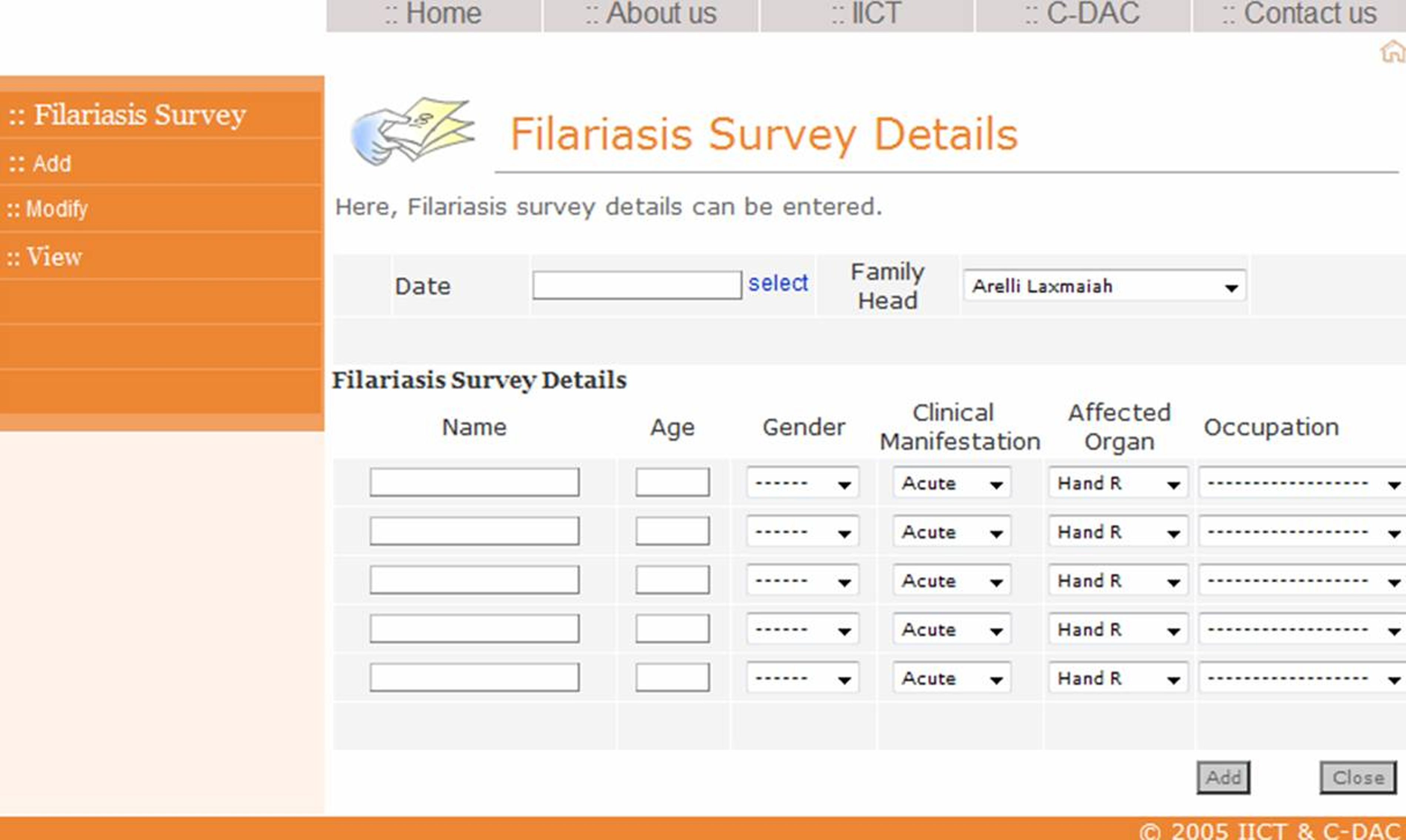

Supplement: Figure S7 — Filariasis disease survey details form. (TIF) [file pone.0039970.s007.tif]

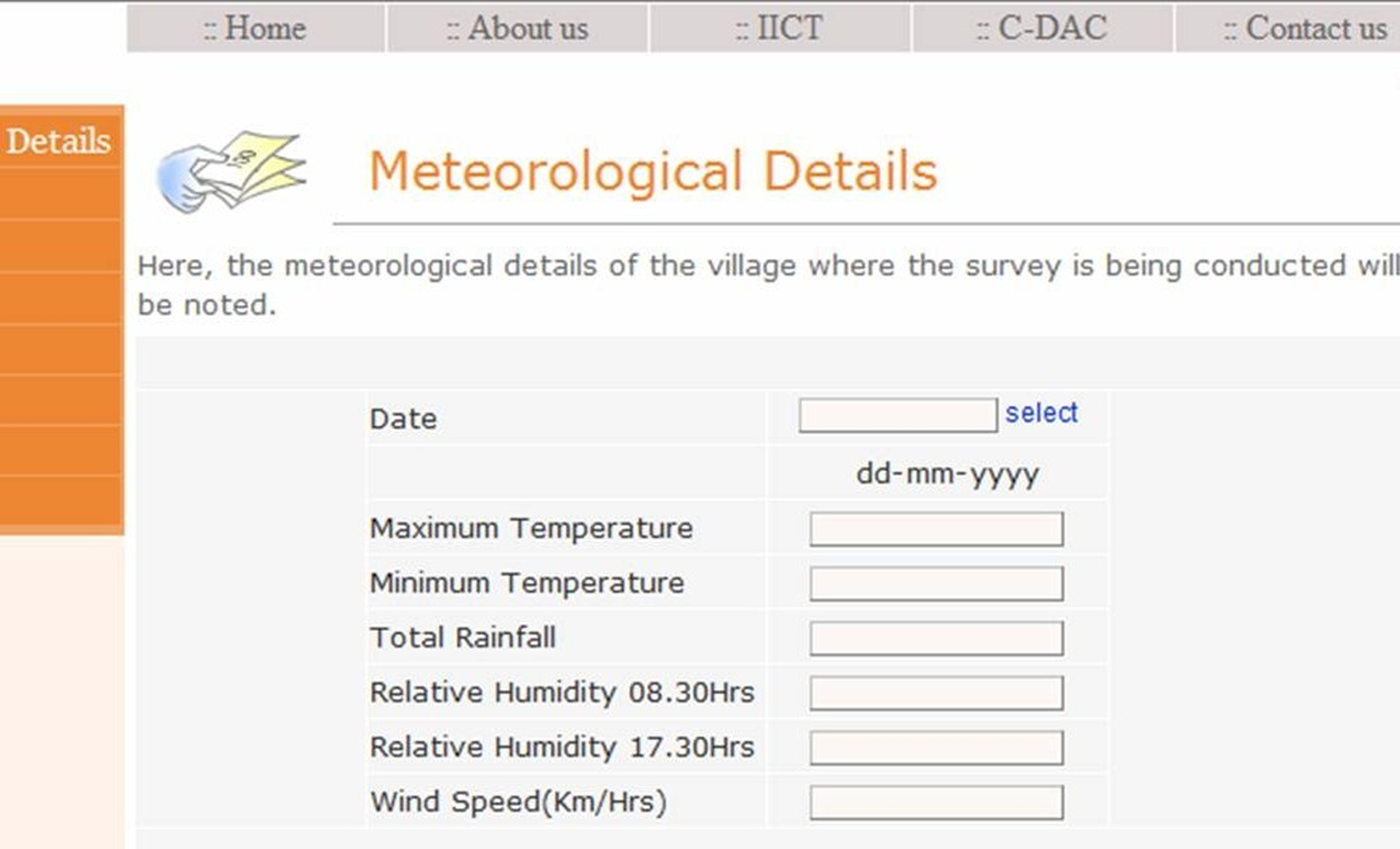

Supplement: Figure S8 — Meteorological details form. (TIF) [file pone.0039970.s008.tif]
